# Supplementary material for: Deep learning reveals that multidimensional social status drives population variation in 11,875 US participant cohort
Source: PLoS One. 2025 Aug 13;20(8):e0327729. doi: 10.1371/journal.pone.0327729 (PMC12349134; doi:10.1371/journal.pone.0327729)
Supplement: S1 File — a) Individual component variance explained. b) Cumulative variance explained across components. S2 Fig. Best CVAE architecture outperforms PCA on reconstruction loss. We compared the mean squared error (MSE) reconstruction loss on the same test split data across 25 different random seed values for PCA solver and CVAE model weight initializations for 6 different CVAE architectures with different hidden layer sizes, all with a 100 dimensional latent space. CVAE architectures 1, 2, and 3 used hidden layer sizes of 200, 400, and 800 respectively. CVAE architectures 4, 5, and 6 first utilized PCA to reduce the data to dimensionality 400, 200, and 150 respectively and had respective hidden layer sizes of 200, 150, and 125. S3 Fig. The top 10 ranked CVAE components account for a high proportion of explained variance. Plotting the CVAE components in order of variance explained lead to the observation that ten components account for a high proportion of variance in the data, indicating that key modes of population stratification exist. S4 Fig. Structure captured by CVAE components predominantly distinct from PCA components. Pearson’s correlation was applied to the top 10 components for both CVAE and PCA ranked by explained variance. The highest correlation is observed between the top PCA component and a range of CVAE components. S5 Fig. Identified SES-centric components predict participant state residency. Confusion matrix reporting the percentage of times state label was correctly predicted across participants (diagonal) versus percentage of incorrect classifications to other states (off-diagonal). S6 Fig. Different SES components are more influential in predicting residency in different US states. Mean logistic regression coefficient values across 10-fold cross validation for each state one-versus-rest classifier. (ZIP) [file pone.0327729.s001.zip › Supporting Information/S2_Table.pdf]

S2 Table

**Category**	**A**	**B**	**C**	**D**	**E**	**F**	**G**	**H**	**I**	**J**
Behavior	16.1	4.5	1.2	2.5	3.3	1	1.7	1.3	6.5	2.3
Cognitive Capacity	19.2	10.2	6.7	9.8	13.3	2.7	7.1	7.5	14.9	12.9
Deep Phenotyping Assessments	18.8	15.2	2.8	3.6	14.7	1.5	3.3	3.8	4.4	1.5
Demographics	27.6	10.7	1.8	8.3	31	3.1	0.6	5.2	10.4	3.4
Diagnostic (KSADS)	15.9	10.2	0.4	4.5	2.4	0.4	0.3	0.3	4.1	0.1
Extracurricular	9.3	8.2	2.5	2.9	24.7	2.7	4.7	3	14.2	3.2
Impulsivity	22.5	21.2	6.2	8.8	7.5	1.2	6.2	1.2	10	0
Life Events	7.8	7.8	2	3.9	0	2	2	3.9	0	17.6
Mania	53.8	0	0	0	11.5	7.7	0	0	0	0
Medical History	4.9	11.3	2	2.6	8.4	1.8	4.1	4.4	3.9	4.4
Medications	21.4	7.1	21.4	0	0	0	0	0	0	0
Mental Health Summary	21.3	0	2.1	0	4.3	0	0	4.3	0	10.6
Neuropsychological Tests	8.3	26	32.4	21.1	12.6	16.8	12.4	19.1	6.1	20.1
Nutrition	5.9	0	5.9	5.9	23.5	0	0	5.9	5.9	0
Parent Characteristics	11.9	7.2	4.6	0.5	10.3	0	2.6	1.5	14.4	3.6
Physical Exam	0	4.7	2.3	7	23.3	0	7	0	7	0

Pubertal Development	18	6	8	2	36	2	2	2	0	2
Sleep	6.1	10.2	1	2	10.7	0.5	3.6	5.6	7.1	0
Social Adjustment	15.4	9.6	4	1.6	26.8	0.7	1.2	2.6	8.8	1.9
Social Responsiveness	25.6	2.3	4.7	2.3	2.3	0	0	0	2.3	0
Socioeconomic	50.5	9.4	2	9.4	38.1	3	1	0	2.5	0
Substance Use	15.4	9.2	3.1	7.4	14.5	6.2	3.1	4.9	9.8	4.9
Trauma	0	11.1	22.2	0	0	0	0	11.1	0	0
