# Supplementary material for: Deep learning reveals that multidimensional social status drives population variation in 11,875 US participant cohort
Source: PLoS One. 2025 Aug 13;20(8):e0327729. doi: 10.1371/journal.pone.0327729 (PMC12349134; doi:10.1371/journal.pone.0327729)
Supplement: Attachment — Submitted filename: REVIEWERS COMMENT_Paper_PONE-D-24-49641.pdf [file pone.0327729.s002.pdf]

REVIEWERS COMMENT

The study highlights the need to see social stratifications—especially those associated with socioeconomic status (SES)—as multifaceted and interrelated with more general social determinants of health. The study recommends that while examining population health and behavior, the entire range of socioeconomic status be taken into account.

Strengths:

- Analyzing a sizable and varied dataset with advanced deep learning (CVAE) gives the results more depth and may reveal patterns that conventional statistical techniques would overlook.
- Because it captures the complexities of social status and how it affects health outcomes, the emphasis on SES as a multifaceted concept is important. This method acknowledges the entire range of variables that interact to influence behavior and health, whereas many studies have a tendency to simplify SES to straightforward metrics like wealth or education.
- Given the increasing emphasis on health equality in public health research, the study emphasizes the need for a sophisticated knowledge of socioeconomic determinants of health.

Limitations:

Although the study placed a lot of emphasis on SES, it might not have taken into consideration other important variables, like genetic impacts or the intricacy of intersectionality (the ways that race, gender, and SES intersect in particular ways).

Conclusion:

All things considered, the study offers insightful information about how population variance is influenced by social status, especially multidimensional SES. It supports the emerging knowledge that health outcomes are intricately linked to both privilege and deprivation, and that resolving these inequities necessitates an appreciation of their complexity.
